# Lehrformate zur Steigerung der Kompetenz von Medizinstudierenden beim Umgang mit Hauterkrankungen bei *Skin of Color* – eine Literaturübersicht

**DOI:** 10.1007/s00105-025-05584-z

**Published:** 2025-09-23

**Authors:** Ariana Fotohi, Inga Hansen-Abeck, Isabel Heidrich, Julian Kött, Stefan W. Schneider, Nina Booken, Finn Abeck

**Affiliations:** Klinik und Poliklinik für Dermatologie und Venerologie, Universitätsklinikum Hamburg-Eppendorf, Martinistraße 52, 20246 Hamburg, Deutschland

**Keywords:** Skin of Color, Hauttyp-Diversität, Medizinstudierende, Dermatologische Lehre, Lehre, Skin of color, Skin type diversity, Medical students, Dermatology education, Teaching

## Abstract

Hauterkrankungen bei Menschen mit Skin of Color (SoC, Hauttyp IV bis VI nach Fitzpatrick) finden in der dermatologischen Ausbildung in Deutschland bisher keine ausreichende Berücksichtigung. Die vorliegende Arbeit zielt darauf ab, den aktuellen Stand zu bisher entwickelten Lehrformaten für Medizinstudierende zur Steigerung der Kompetenz beim Umgang mit Hauterkrankungen bei SoC aufzuzeigen. In einer Literaturrecherche mit vordefinierten Kriterien in PubMed MEDLINE wurden 7 Studien eingeschlossen. Der Großteil der Lehrformate wurde auf freiwilliger Basis angeboten. Die Anzahl der Teilnehmenden reichte von 20–172. Unser Review verdeutlicht, dass Interventionen zu SoC in der dermatologischen Lehre sowohl die diagnostische Kompetenz als auch das subjektive Vertrauen in die eigenen Fertigkeiten von Medizinstudierenden verbessern können. Diese Übersichtsarbeit kann als Ausgangspunkt für die stärkere Integration einer diversitätssensiblen Lehre im Rahmen der medizinischen Ausbildung genutzt werden.

## Abstract (en)

Skin diseases affecting people with skin of color (SoC; Fitzpatrick skin types IV–VI) have not been given adequate attention in dermatological training in Germany. This study aims to present teaching formats developed to improve medical students’ skills in managing these diseases. A literature search was conducted in PubMed Medline using predefined criteria, which resulted in seven studies being included. Most of the teaching formats were offered on a voluntary basis, with participant numbers ranging from 20 to 172. Our review shows that interventions focusing on SoC in dermatological education can enhance medical students’ diagnostic abilities and subjective confidence in their skills. This overview may serve as a foundation for further integrating diversity-sensitive teaching into medical education.

## Einleitung

Im Gesundheitssektor gewinnt die ethnische Zugehörigkeit zunehmend an Bedeutung, insbesondere im Hinblick auf die Tatsache, dass ein Großteil der Weltbevölkerung und somit auch der Patient:innen dunklere Hauttypen aufweist [7, 13]. Nach Fitzpatrick können in Abhängigkeit des Sonnenbrand- und Bräunungsverhaltens sechs verschiedene Hauttypen unterschieden werden, die von Hauttyp I (sehr helle Haut) bis Hauttyp VI (dunkelbraune bis schwarze Haut) reichen [4]. Menschen mit Skin of Color (SoC) können durch die Hauttypen IV–VI charakterisiert werden [17].

Trotz der demographischen Entwicklung in vielen Ländern hin zu ethnisch vielfältigeren Bevölkerungen ist die Abbildung von Patient:innen mit SoC in der medizinischen Ausbildung in Deutschland nach wie vor unzureichend [3]. Diese unausgewogene Darstellung, bei der Menschen mit SoC unterrepräsentiert sind, findet sich auch in deutschen Lehrbüchern wieder [3]. Insbesondere bei der Beurteilung von Hauterkrankungen bei SoC besteht unter Dermatolog:innen in Deutschland sowie weltweit weiterhin eine diagnostische Unsicherheit [2]. Dies unterstreicht die Notwendigkeit, gezielte Programme zur Verbesserung der diagnostischen Fähigkeiten im Umgang mit Hauterkrankungen bei SoC zu etablieren [2].

Strukturelle Defizite in der Ausbildung, wie das Fehlen von diversitätsorientierten Inhalten innerhalb der Curricula, beeinträchtigen auch die diagnostische Kompetenz der Studierenden sowie Fachkräften und könnten auch zukünftig zur Versorgungsungleichheit beitragen [9, 12]. In den letzten Jahren wurden durch das zunehmende Bewusstsein für die Unterrepräsentation von SoC in der Medizin eine Reihe von verschiedenen Lehrmethoden entwickelt, um diese Lücke in der Dermatologie zu schließen [11, 18].

Die vorliegende Arbeit zeigt den aktuellen Stand zu bisher entwickelten Lehrformaten für Medizinstudierende zur Steigerung der Kompetenz beim Umgang mit Hauterkrankungen bei SoC auf.

## Methodik

Es erfolgte eine Literaturrecherche in der Datenbank MEDLINE via PubMed im Mai 2025. Hierfür wurde genutzt: „skin of color“ AND („curriculum“ OR „course“ OR „students“ OR „education“). Als Einschlusskriterien festgelegt wurden (1) englische und deutschsprachige Publikationen, die (2) Hauterkrankungen bei SoC thematisieren, (3) eine gezieltes Lehrformat zur Steigerung der Kompetenz beinhalten und (4) Medizinstudierende einschließen. Arbeiten, die keine Intervention beinhalten oder lediglich Ärzt:innen oder Pflegende als Studienpopulation untersuchen, wurden ausgeschlossen. Die Recherche ergab insgesamt 235 Treffer. Nach Titel- und Abstract-Screening wurden 201 Titel ausgeschlossen. Nach Prüfung der Volltexte auf Eignung wurden von den verbleibenden 34 Arbeiten schließlich 7 Publikationen eingeschlossen (12 Publikationen wurden ausgeschlossen, da keine Medizinstudierenden untersucht wurden, 15 Publikationen wurden ausgeschlossen, da kein gezieltes Lehrformat untersucht wurde).

## Ergebnisse

Die Studiencharakteristika mit ihren wesentlichen Merkmalen sind in Tab. 1 dargestellt. Von den 7 eingeschlossenen Arbeiten wurden 3 in den USA [5, 8, 15], 2 in Kanada [10, 14] durchgeführt. Eine Arbeit stammte aus Deutschland [1], eine aus Botswana [16]. Die Anzahl der Teilnehmenden reichte von 20–172 Personen, die Studien wurden zwischen 2021 und 2025 publiziert.

**Tab. 1:** Veröffentlichungen zu Lehrformaten für Medizinstudierende zu Hauterkrankungen bei *Skin of Color (SoC)*

Autor (Land)	Studierende	Lehrformat	Ergebnisse
Seeburruth et al. [14] (Kanada)	*n* = 172	Digitales Selbstlernmodul zu SoC	Verbesserung der diagnostischen Fähigkeiten bei Dermatosen bei SoC (MC-Test)
		Themen: Hauterkrankungen, die häufig bei SoC vorkommen	Verbesserung des eigenen Vertrauens bei der Diagnose von Dermatosen bei SoC gemäß Selbsteinschätzung
		Freiwillige Teilnahme	
Abeck et al. [1] (Deutschland)	*n* = 158	Seminar in Präsenz	Steigerung des Wissens über die Präsentation von Dermatosen bei SoC und damit einhergehenden psychosozialen Belastungen gemäß Selbsteinschätzung
		Dauer: 90 min (davon 45 min zu SoC)	Verbesserung der Diagnosefähigkeit bei Dermatosen bei SoC gemäß Selbsteinschätzung
		Themen: Hauttypdiversität	
		Pflichtveranstaltung in der Regellehre mit Anwesenheitserfassung	
Han et al. [5] (USA)	*n* = 43	Digitale Vorlesung	Vor der Teilnahme: geringere Diagnosefertigkeit für Dermatosen bei SoC im Vergleich zu hellerer Haut (MC-Test)
		Dauer: 60 min	Verbesserung der Diagnosefertigkeit sowohl bei hellerer als auch bei dunklerer Haut (MC-Test)
		Themen: Hauterkrankungen, die häufig im amerikanischen Staatsexamen geprüft werden mit Fokus auf Erkrankungen bei SoC	Verbesserung der selbsteingeschätzten Diagnosefertigkeit für Dermatosen bei SoC
		Freiwillige Teilnahme	
Kojder et al. [8] (USA)	*n*= 36*	Digitale Vorlesung	Verbesserung der diagnostischen Genauigkeit für Dermatosen bei SoC (MC-Test)
		Dauer: 60 min	Verbesserung des eigenen Vertrauens bei der Beurteilung von Dermatosen bei SoC gemäß Selbsteinschätzung
		Themen: Präsentation von Hauterkrankungen auf hellerer und dunklerer Haut	Positive Effekte auch 3 Monate nach der Teilnahme vorhanden
		Freiwillige Teilnahme	
Mardiros et al. [10] (Kanada)	*n*= 20	Digitales Selbstlernmodul zu SoC	Verbesserung der diagnostischen Fähigkeiten bei Dermatosen bei SoC (MC-Test)
		Freiwillige Teilnahme	
Shango et al. 15] (USA)	*n* = 77	Digitales Selbstlernmodul zu SoC	Steigerung des Vertrauens in die eigene diagnostische Kompetenz bei Dermatosen bei SoC gemäß Selbsteinschätzung
		Themen: Hauterkrankungen, die häufig bei SoC vorkommen	
		Freiwillige Teilnahme	
Slaught et al. [16] (Botswana)	*n* = 57	Digitales Selbstlernmodul zu SoC	Verbesserung der diagnostischen Genauigkeit und der Geschwindigkeit (MC-Fragen)
		Dauer: im Mittel 2 h	Stärkung des Selbstvertrauens in Diagnose und Beschreibung von Hauterkrankungen gemäß Selbsteinschätzung
		Themen: Hauterkrankungen, die häufig bei SoC vorkommen	
		Freiwillige Teilnahme	

*SoC* Skin of Color, *MC-Test* Multiple-Choice-Test

^*^In der Teilnehmendenzahl sind neben Medizinstudierenden auch Assistenzärzt:innen, Fachärzt:innen, Physician Assistants und Pflegekräfte enthalten

Seeburruth et al. [14] konzipierten im Rahmen eines nationalen Pilotprojekts an allen medizinischen Fakultäten Kanadas 10 webbasierte Lernmodule, die über *Social-Media* zugänglich waren und freiwillig bearbeitet werden konnten. Über die jeweilige Dauer der Module oder die Gesamtbearbeitungszeit gibt es keine Angaben. Die Module umfassten bei Menschen mit SoC häufig vorkommende Hauterkrankungen. Vor und nach den Modulen sollten die Teilnehmenden einen Fragebogen ausfüllen, der über objektive Messinstrumente (Multiple-Choice-[MC]-Fragen) klinisches Wissen und über subjektive Messinstrumente (Selbsteinschätzung) die persönlich wahrgenommene diagnostische Sicherheit überprüfte. Insgesamt nahmen 172 Medizinstudierende teil. Nach Abschluss der Module ließ sich eine deutliche Zunahme des Fachwissens und des Selbstvertrauens in die diagnostische Sicherheit bezüglich Dermatosen bei SoC feststellen. Mehr als 95 % der Studierenden befürworteten eine curriculare Integration der Module.

Abeck et al. [1] implementierten ein 90-minütiges interaktives Seminar zur Hauttypdiversität in das 7. Semester der Regellehre am Universitätsklinikum Hamburg-Eppendorf (Deutschland). Die Seminare fanden jeweils als Präsenzveranstaltung mit Anwesenheitspflicht statt. Es wurden Hauterkrankungen auf unterschiedlichen Hauttypen präsentiert und psychosoziale Aspekte bei der Behandlung von Dermatosen gelehrt. Vor und nach dem Seminar füllten die Studierenden einen Fragebogen zur selbsteingeschätzten Kompetenz aus. Es nahmen 162 Studierende teil, von denen 158 in die Auswertungen eingingen. Nach Absolvierung des Seminars ließen sich eine Zunahme des Wissens über die Ausprägung von Hauterkrankungen bei Menschen mit SoC und damit einhergehende psychosoziale Belastungen, über Häufigkeitsunterschiede von Hauterkrankungen bei unterschiedlichen Hauttypen und der Fähigkeiten einer Diagnosestellung von Hauterkrankungen bei dunkleren Hauttypen gemäß Selbsteinschätzung der Studierenden feststellen. Der Großteil der Teilnehmenden gab an, sich mehr Veranstaltungen zu diesem Thema zu wünschen.

Han et al. [5] entwickelten an der Icahn School of Medicine at Mount Sinai (New York City, NY, USA) ein virtuelles Lehrmodul auf freiwilliger Basis, das aus einer einstündigen Vorlesung bestand. Die Vorlesung umfasste mehr als 30 Hauterkrankungen auf unterschiedlichen Hauttypen und basierte auf Prüfungsinhalten des amerikanischen Staatsexamens mit häufig fehldiagnostizierten dermatologischen Erkrankungen bei SoC. Zur Messung der Effektivität des Lehrmoduls wurden objektive (MC-Tests mit Blickdiagnosen) und subjektive Messinstrumente (Selbsteinschätzung) eingesetzt. Es nahmen 46 Studierende an der Veranstaltung teil, von denen 43 in die Auswertungen eingingen. Im objektiven Vortest (MC-Fragen) zeigten die Studierenden eine höhere Diagnosesicherheit für Dermatosen auf hellerer Haut im Vergleich zu SoC. Nach dem Seminar zeigte sich sowohl für Erkrankungen bei SoC als auch auf hellerer Haut eine Verbesserung der studentischen Diagnosefähigkeit (30,1 und 25,6 %). Auch die selbsteingeschätzte Kompetenz zu Hauterkrankungen bei SoC verbesserte sich nach der Teilnahme.

Kojder et al. [8] erarbeiteten an der Dell Medical School (University of Texas, USA) ein auf freiwilliger Mitarbeit beruhendes einstündiges Online-Modul, das aus einer Vorlesung, einem Vortest und einem Nachtest sowie im späteren Verlauf einem 3‑Monats-Follow-up-Test bestand. Die Teilnehmenden bestanden aus einer heterogenen Gruppe aus Medizinstudierenden, Assistenzärzt:innen, Fachärzt:innen, Physician Assistants und Pflegekräften. Inhaltlich wurden 20 dermatologische Krankheitsbilder auf heller und dunkler Haut anhand von Abbildungen, Erklärungstexten und Diagnosehilfen vermittelt. Die Effektivität des Moduls wurde objektiv (MC-Tests mit Blickdiagnosen) und subjektiv (Selbsteinschätzung des Diagnosevertrauens) erfasst. Von den 100 Teilnehmenden, die den Vortest abgeschlossen haben, nahmen 36 auch am Follow-up 3 Monate später teil. Die diagnostische Genauigkeit und das eigene Vertrauen verbesserten sich nach der Intervention signifikant sowohl für Erkrankungen auf hellerer als auch auf dunklerer Haut. Drei Monate später zeigte sich weiterhin eine Verbesserung der Diagnoseleistung für Dermatosen bei SoC.

Mardiros et al. [10] integrierten ein digitales Selbstlernmodul, Skin of Colour Dermatoses Self-Learning Module, in das dermatologische Curriculum des zweiten Studienjahres des Fachbereichs Medizin an der University of Ottawa, Ontario (Kanada), das seither ein fester, wenngleich freiwilliger Bestandteil der Lehre ist. Über Dauer und Inhalt des Moduls werden im Rahmen der Publikation keine Angaben gemacht. Vor und nach dem Modul wurden die Studierenden aufgefordert, einen didaktisch und bildbasiert aufgebauten Fragebogen auszufüllen, der Dermatosen adressierte, die bei SoC häufiger vorkommen oder einen schwereren Verlauf zeigen. Der Wissenszuwachs wurde objektiv (MC-Tests) im Prä-post-Design erfasst. Es nahmen 25 Studierende an dem Modul teil, von denen 20 in die Auswertung eingeschlossen wurden. Nach dem Modul zeigte sich die Antwortrate an korrekt gestellten Diagnosen in Bezug auf Dermatosen bei SoC im Mittel deutlich verbessert.

Shango et al. [15] implementierten im Rahmen des Kurses „Musculoskeletal, Dermatology, and Peripheral Nervous System“ ein interaktives E‑Learning-Modul zum Thema SoC in das zweite Studienjahr der Wayne State University School of Medicine (Detroit, MI, USA). Zur Dauer des Moduls wurden keine Angaben gemacht. Die Lehrintervention wurde optional am Ende des Kurses online angeboten. Im Rahmen des Moduls wurden 13 Dermatosen, die typischerweise bei SoC vorkommen, fallbasiert vermittelt. Vor und nach dem Modul wurde jeweils mittels Fragebogen die Selbsteinschätzung des Vertrauens in die Diagnosefähigkeit auf subjektiver Basis untersucht. Von den 145 Studierenden, die den Prätest begonnen haben, beendeten 77 das gesamte Modul inklusive Posttest. Das Selbstvertrauen in die diagnostischen Fähigkeiten bezüglich des Erkennens von Dermatosen bei SoC zeigte sich im Nachtest deutlich gesteigert. Die Mehrheit der teilnehmenden Studierenden bewertete das Modul als hilfreich und forderte eine breitere Integration des Themas in das Curriculum.

Slaught et al. [16] entwickelten an der University of Botswana (Gaborone, Botswana) ein digitales und optionales Selbstlernmodul im Rahmen einer verpflichtenden klinischen Dermatologierotation im vierten und fünften Studienjahr. Die durchschnittliche Bearbeitungszeit der Module betrug unter 2 h. Die Module umfassten 4 eigenständige, bildbasierte PALMs (Perceptual and Adaptive Learning Modules). Inhaltlich wurden häufige dermatologische Erkrankungen auf SoC dargestellt und durch wiederholtes, adaptives Lernen visuell eingeprägt. Die Wirksamkeit wurde sowohl objektiv durch Vor- und Nachtests sowie einem verzögertem Nachtest (MC-Fragen) mit Abbildungen dermatologischer Erkrankungen als auch auf subjektiver Ebene (Selbsteinschätzung) ermittelt. Insgesamt gingen die Ergebnisse von bis zu 57 (je nach PALM) Medizinstudierenden in die Auswertung ein. Nach der Durchführung der Module verbesserte sich die diagnostische Genauigkeit signifikant. Die Studierenden beurteilten die Module in einer Befragung überwiegend als hilfreich, praxisnah und stärkend für ihr Selbstvertrauen in Diagnose und Beschreibung von Hauterkrankungen.

## Diskussion

Im Rahmen dieses Reviews wurden verschiedene Lehrformate untersucht, die allesamt das Ziel verfolgen, die Kompetenz von Medizinstudierenden im Bereich dermatologischer Erkrankungen bei Patient:innen mit SoC zu verbessern. Nach jeder der 7 dargestellten Publikationen konnte diese Kompetenz mithilfe gezielter Bildungsangebote verbessert werden. Lediglich eine Lehrveranstaltung fand hierbei als Präsenzunterricht [1] statt, während die übrigen Formate digital als Vorlesung [5, 8] oder Selbstlernmodul [10, 14–16] konzipiert wurden.

Einen zentralen Aspekt der vorgestellten Publikationen stellt die Freiwilligkeit der Teilnahme an den Lehrinterventionen dar. Während der Großteil der Formate auf freiwilliger Basis erfolgte [5, 8, 10, 14–16], wurde lediglich ein Modul verpflichtend in das Curriculum integriert [1]. Dabei lassen sich positive Effekte über die Wirksamkeit der einzelnen Interventionen in beiden Gruppen nachweisen. Verpflichtende Lehrveranstaltungen können in einer hohen Teilnahmequote resultieren, die in freiwilligen Lehrangeboten deutlich niedriger ausfällt. Die curriculare Einbettung scheint daher weniger ausschlaggebend für den kurzfristigen Lernerfolg zu sein, jedoch könnte die systematische Verankerung der Intervention langfristig nachhaltiger wirken. Zudem richten sich freiwillige Formate häufiger primär an bereits dermatologisch interessierte Studierende, was zu einem Selektionsbias führen kann und zur Folge hat, dass Studierende ohne Vorinteresse kaum erreicht werden und somit eine breitere Sensibilisierung für das Thema Hauterkrankungen bei SoC ausbleibt [6].

Bei manchen Lehrinterventionen lag die Gesamtdauer zwischen einer Stunde [5, 8] und 90 min [1], während sie bei einer anderen, die modular aufgebaut war, eine längere Bearbeitungszeit von ca. 2 h erforderte [16]. Wieder andere Studien machten keine Angaben zu der Dauer der Lehrformate [10, 14, 15]. Die Ergebnisse zeigen, dass auch kurze Lehrinterventionen signifikante Verbesserungen der diagnostischen Fähigkeiten oder der subjektiven Wahrnehmung erbringen konnten. Umfangreichere Formate hingegen ermöglichen eine vertiefte Auseinandersetzung mit SoC in der Dermatologie und können einen nachhaltigeren Lerneffekt bieten, der meist aber mit höherem Ressourcenaufwand verbunden ist.

Die Erhebung der Effektivität erfolgte in den untersuchten Studien methodisch heterogen. Während einige Arbeiten sowohl objektive als auch subjektive Erhebungsinstrumente kombinierten [8, 14, 16], beschränkten sich andere entweder ausschließlich auf Selbsteinschätzung [1, 15] oder auf objektive Messverfahren wie MC-Fragen [5, 10]. Subjektive Verfahren geben einen Einblick in die wahrgenommene Selbsteinschätzung der Studierenden, jedoch sind diese anfällig für Verzerrungen, wie soziale Erwünschtheit oder den Hawthorne-Effekt (Veränderung des Verhaltens im Rahmen von Studienteilnahmen). Der Einsatz objektiver Messverfahren, wie bildbasierte MC-Tests, ermöglicht eine differenziertere Bewertung des tatsächlichen Wissens- und Kompetenzzuwachses und ist damit besonders relevant für die Validierung von Lehrinterventionen.

Eine weitere Limitation liegt bei der fehlenden Überprüfung der Langzeitergebnisse. Die Mehrheit der Studien beschränkte sich auf unmittelbare Prä-post-Vergleiche [1, 5, 10, 14, 15], durch welche keine Rückschlüsse auf die Nachhaltigkeit der Lerneffekte möglich sind. Lediglich 2 Studien integrierten Follow-up-Befragungen nach mehreren Wochen bzw. Monaten [8, 16]. Diese konnten nachhaltige Verbesserungen in Wissen und Diagnosefähigkeit nachweisen.

Unser Review verdeutlicht, dass Lehrformate zu SoC in der Dermatologie sowohl die diagnostische Kompetenz als auch das subjektive Vertrauen in die eigenen Fertigkeiten von Medizinstudierenden verbessern können, wobei sich auch kürzere und gezielte Interventionen als wirksam erwiesen haben. Es wäre wünschenswert, dass – neben der Entwicklung weiterer Lehrkonzepte zu SoC – zukünftige Lehrformate verpflichtend in die medizinischen Curricula implementiert werden, um der freiwilligen Selbstselektion entgegenzuwirken und eine breitere Sensibilisierung für dieses hochrelevante Thema zu ermöglichen. Zur Validierung der Wirksamkeit der Ergebnisse empfiehlt sich der Einsatz von kombinierten Messverfahren. Darüber hinaus sind Langzeiterhebungen notwendig, um die Nachhaltigkeit der Lerneffekte besser beurteilen zu können. Unsere Literaturübersicht kann als Ausgangspunkt für die vermehrte Integration einer diversitätssensiblen Lehre im Rahmen der medizinischen Ausbildung genutzt werden.

## Fazit für die Praxis

- Hauterkrankungen bei *Skin of Color* (SoC) sollten zukünftig in der medizinischen Ausbildung stärker thematisiert werden.
- Mithilfe von Lehrveranstaltungen können bereits im Medizinstudium das Wissen über Dermatosen bei *SoC* und damit die entsprechende Diagnosefähigkeit gefördert werden.
- Untersuchungen zu Langzeiteffekten auf die Fertigkeiten der Studierenden sollten zukünftig durchgeführt werden.
